# Magnetic nanofluid behavior including an immersed rotating conductive cylinder: finite element analysis

**DOI:** 10.1038/s41598-021-83944-0

**Published:** 2021-02-24

**Authors:** Hameed K. Hamzah, Farooq H. Ali, M. Hatami, D. Jing, Mohammed Y. Jabbar

**Affiliations:** 1 College of Engineering -Mechanical Engineering Department, University of Babylon, Babylon City, Hilla Iraq; 2 International Research Center for Renewable Energy, State Key Laboratory of Multiphase Flow in Power Engineering, Xi’an Jiaotong University, Xi’an, 710049 China; 3 Department of Mechanical Engineering, Esfarayen University of Technology, Esfarayen, North Khorasan Iran

**Keywords:** Chemical engineering, Energy infrastructure, Mechanical engineering

## Abstract

In this paper, numerical Galerkin Finite Element Method (GFEM) is applied for conjugate heat-transfer of a rotating cylinder immersed in Fe_3_O_4_-water nanofluid under the heat-flux and magnetic field. The outer boundaries of the cavity were maintained at low temperatures while beside the cylinder were insulated. It is assumed that the cylinder rotates in both clockwise and counter-clockwise directions. The dimensionless governing equations such as velocity, pressure, and temperature formulation were analyzed by the GFEM. The results were evaluated using the governing parameters such as nanoparticles (NPs) volume fraction, Hartmann and Rayleigh numbers, magnetic field angle and NPs shapes. As a main result, the average Nusselt number increases by increasing the NPs volume fraction, inclination angle and thermal conductivity ratios, while increasing the Hartmann number decreased the Nusselt number. Furthermore, platelet NPs had the maximum average Nusselt number and spherical NPs made the minimum values of Nusselt numbers among examined NPs shapes.

## Introduction

Rotating cylinders in the fluids due to their wave production and vortex generations have a large application in heat-transfer processes, industrial engineering’s, electrical productions, chemical engineering, etc. Recently, researchers focused on the rotating cylinders behavior in the fluid media. For example, Selimefendigil and Öztop^1^ investigated the effect of rotating cylinder on the phase change material (PCM) heat-transfer in a square cavity using numerical analysis. A maximum value of the heat-transfer for different perpendicular locations of the cylinder depends on the angular rotational velocity of cylinder. The effect of radius and rotational velocity of the cylinder has been considered by Costa and Raimundo^2^. Recently, Hussain et al.^3^ investigated the forced convection of rotating cylinder in a horizontal channel by finite element method (FEM), wherein a clockwise rotation of the cylinder creates the fluid flows over the cylinder, while the fluid flows below the cylinder in the anticlockwise. Kumar et al.^4^ studied the effect of Reynolds and Prandtl numbers on a confined semi-circular cylinder for the vortex producing. In a three dimensional numerical analysis, Selimefendigil and Öztop^5^ studied conjugate heat-transfer of rotating cylinder in a cubic cavity filled by CNT (carbon nanotube) -water nanofluid similar to a 3D study performed in a cubic cavity^6^.

In a different study, Zhuang et al.^7^ eaxamined the effect of a wavy cylinder on the downstream flow using finite volume method and found a distinct difference in the vortex structures between a rotating wavy cylinder and a stationary wavy cylinder, which confirms the results of vortices produced by the wavy cylinder^8^. Evidently, cylinders have significant effect on the heat- transfer due to vortex generations, so having two or more cylinders on the flow may effect on each other and flow patterns. Khanafer et al.^9^ studied this effect of two cylinders on mixed convection heat-transfer in a partially heated cavity, illustrating that the magnitude and direction of the rotation speed of the cylinders have a significant effect on the flow pattern, isotherms and Nusselt numbers. Moreover, Lacroix^10^ had noticed the effect of two cylinder on the natural convection heat-transfer in a horizontal cavity a long before. Zhang et al.^11^ illustrated the unsteady mixed convective heat-transfer between a square enclosure and an inner impulsively rotating cylinder that when initial velocity increases, the local Nusselt number has more noticeable sequential variation at the left and bottom sidewalls of the enclosure, as also claimed by Fu et al.^12^, confirming rotation direction of the cylinder is critical for this specific configuration and has significant effect on the outcomes. An entropy generation study on the heated rotating cylinder inside a flexible wall cavity reveals the highest entropy generation rates for a counter-clockwise rotation of the circular cylinder^13^. The studies were extended on a wavy-walled cavity filled with nanofluid and involving a rotating cylinder^14^. Sasmal et al.^15^ studied the effect of rotating cylinder submerged in a power-law fluid, with a new correlation for Nusselt number based on Grashof, Rayleigh, Prandtl, rotational velocity and other related parameters. On the other hand, a similar study on the power-law flow including two heated cylinders by Mishra et al.^16^ reports the same behavior of heated cylinders. Studies of the effect of magnetic field on the forced convection of ferrofluid including a rotating cylinder report that the electromagnetic force slows down the ferrofluid flow, but the drag coefficient is enhanced^17,18^.

Using a nanofluid is another efficient way to enhance heat-transfer for cooling processes, so wide studies are considered to improving their performance or extend their applications by optimization techniques. In an experimental study, Song et al.^19^ predicted a precious formulation of TiO_2_-water properties. Tang et al.^20^ investigated nanofluid flow in a double sinusoidal wavy cavity and discussed on the Nusselt numbers variations by Rayleigh numbers. Zhou et al.^21^ considered an application of wavy walls in a nanofluid-filled microchannel by optimized geometry parameters to reach better heat-transfer. Ghadikolaei et al.^22^ studied effect of magnetic field in alcoholic based nanofluid in a porous medium and its treatments. Furthermore, Hatami et al.^23–26^ applied the optimization techniques to improve the geometries and enhance the nanofluid heat- transfer in applications such as solar collectors. Farooq et al.^27^ studied Cu-water nanofluid flow in an annulus enclosure with inner rotating corrugation cylinder of an average Nusselt number can be improved by increasing the Rayleigh number. Also, Alsabery et al.^28^ studied Al_2_O_3_-water nanofluid in double lid-driven square cavity using two-phase method.

In general, most of the studies are focused on heat-transfer in geometrical cavities due to its applications. Mixed convection studied in a lid-driven parallelogram-shaped enclosure duly affects vital parameters such as Richardson number on the Nusselt number and skin friction coefficient^29^. Ismael^30^ studied mixed convection in a cavity with arc-shaped moving wall in contest to the Rayleigh effect on the heat transfer. Studies of effect of non-concentric position of a rotating cylinder in a square cavity reveal that the bottom left corner is the best position of rotating cylinder in view of the heat-transfer^31,32^. A numerical analysis of the effects of magnetic field on fluid flow and heat-transfer in two-dimensional square cavity implies the recirculation eddy is reduced in the cavity in magnetic field^33^. A mixed convection of air-filled cavity considering two sinusoidal wall suggests a design of orthogonal sinusoidal walls gives a higher heat-transfer over the vertical and horizontal sinusoidal walls^34^. Yang et al.^35^ examined time-periodic combined natural-forced (mixed) convection in a cold square enclosure walls having hot rotating circular cylinder at high Rayleigh number, Ra = 10^6^. This work inspired to investigate the unsteady periodic of rotating circular cylinder and its effect on temperature distribution and fluid structure, in the cylinder rotation reduces the heat-transfer rate^36^.

Hussain et al.^37^ studied entropy in mixed convection in a horizontal channel of a rectangular open enclosure and a square obstacle. The channel was filled with an Al_2_O_3_-Cu-water nanofluid and a magnetic force was applied horizontal to the cavity. Heat and mass-transfer were studied in a porous medium filled with three nanofluids (Cu, Al_2_O_3_, TiO_2_) under the effect of magnetic field, thermal radiation, viscous dissipation and chemical reaction^38^. Also, the mixed convection in a square enclosure lid-driven having top and bottom moving walls under the effect of the inclined magnetic field was studied^39^. In these studies, Al_2_O_3_-water nanofluid was chosen as a working fluid and sinusoidal function was applied to the hot left side wall, keeping right wall at low temperature. The inclination angle and Reynolds number have a large effect on fluid flow and heat-transfer rate. Hussain et al.^40^ examined magnentohydrodynamics and entropy in mixed convection of lid-driven T-shaped porous cavity in Galerkin FEM, with parameters Richardson number (Ri), Darcy number (Da), angle of inclination of magnetic field (γ), aspect ratio (AR) and Hartmann number (Ha). A numerical study was performed of natural convection inside open cavity filled with porous-nanofluid as a two-phase mode^41^. In the partial differential equations solved in the Galerkin FEM, the heat-transfer rate is affected by thermophoresis parameter and Brownian motion. Further, a numerical study was extended for a two-dimensional impingement flow of SiO_2_-water nanofluid^42,43^. The bottom wall was hot and cooled by the jet flow from the top wall. They compared the results of flat and corrugated hot bottom walls with the parameters as Reynolds number, amplitude and frequency of corrugation wall, volume of fraction and shapes of nanoparticles. A corrugated wall gives a better heat-transfer rate than flat wall. Further, an adiabatic rotating cylinder in the middle distance between top and bottom walls was inserted to control over heat-transfer rate. In the results, average Nusselt number decreases as rotation speed rises, but it aroused as the volume fraction had increased. Dogonchi et al.^44^ have explored natural magneto-hydrodynamic CuO-water nanofluid in a complex geometry using controled volume FEM for solving the equations of continuity, momentum and energy. It was found that the heat-transfer rate had increased as the Rayleigh number increased, but decreased on the Ha increasing. Also, it was reported that platelet nanoparticles (NPs) had the greatest performance compared to other shapes. Mixed convection was studied in porous U-shaped channel with 3D geometry and two rotating cone^45^, and that of nanofluid in double steps of forward facing with four rotating circular cylinders under the effect of magneto-hydrodynamic was examined^46^. Altought the above litreatures focused on the application of nanofluids, but studying on the rheology of different nanofluids is also very important to can find the reasons of nanofluid behaviors in different applications. Susruth et al.^47^ investigated the rheology of nanofluids when using gold nanoparticles as additives. Also, Phule et al.^48^ and Susrutha et al.^49^ focused on the poly molecules effects on rheology and stability of nanofluids, correspondingly.

There are many engineering and industrial applications where the thermal heat flux is the main and effective boundary condition. Therefore, consideration was given to the effect of thermal heat flux on upper half of the rotating cylinder in the present work. Also, the papers published in the recent years have been focused on the influence of the magnetic field on fluid flow and heat transfer in nanofluids due to its importance in controlling the behavior of fluid. Evidently, conjugate mixed convection heat-transfer has many usages, namely, solar collector, heat exchanger, radiator engines cooling systems, condenser and evaporator of cooling and heating systems, asphalt paving, rolling and metal forming processes, etc. Most of the researches were focused on constant wall temperature boundary condition (constant cold and hot temperature), while current work, versus the previous studies, considered the constant heat flux on a curved cylindrical wall in addition to a constant straight walls temperature. Also, This work provided a numerical model for a rotating cylinder in two different spaces and the changes that the cylinder underwent due to the different boundary conditions, thermal heat flux from the top and the thermal conjugate heat transfer from the bottom. All of these assumptions were occurring under magnetic field effect. Furthermore, the effects of the nontrivial parameters on the streamline, temperature contours and Nusselt numbers are discussed.

## Mathematical modeling

**Figure 1:**
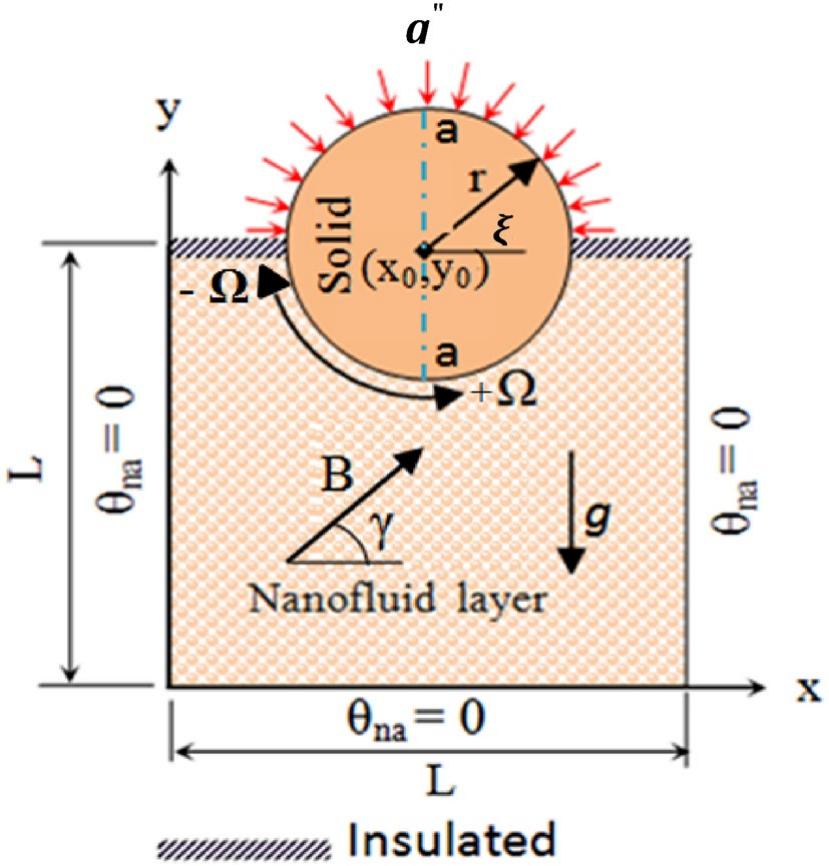
Schematic diagram of the present problem.

A two-dimensional model—a conjugate system of solid conductive rotating cylinder partly immersed in a Fe_3_O_4_ - nanofluid is considered as shown in Fig. 1. Fe_3_O_4_ NPs with high thermal and magnetic properties are chosen to make a magnetic-fluid due to its cost effective values. It is assumed no heat generation or absorption occurs and viscous dissipation and Joule-heating effects are ignored in absence of any chemical reactions. Upper surface of the conductive rotating cylinder is exposed to constant heat-flux (${\varvec{q}}^{\prime \prime }$), and the bottom and two vertical walls are kept at cold temperature (T_c_). The center of rotation of the cylinder is (X_o_, Y_o_) with three conditions speed of rotation, Ω = 1000, 0, (−) 1000. The domain varies from top of rotating conductive cylinder to its cold bottom wall. Thermal properties of the nanofluid are described in Table 1, with different shapes of NPs given in Table 2. The heat-capacity, density, thermal diffusivity and thermal expansion of nanofluid can be calculated with equations;

1 $$\rho_{na} = \left( {1 - \emptyset } \right)\rho_{bf} + \emptyset \rho_{sp} ,$$

2 $$\left( {\rho c_{p} } \right)_{na} = \left( {1 - \emptyset } \right)\left( {\rho c_{p} } \right)_{bf} + \emptyset \left( {\rho c_{p} } \right)_{sp } ,$$

3 $$\alpha_{na} = \frac{{k_{na} }}{{\left( {\rho c_{p} } \right)_{na} }},\quad and$$

4 $$\left( {\rho \beta } \right)_{na} = \left( {1 - \emptyset } \right)\left( {\rho \beta } \right)_{bf} + \emptyset (\rho \beta )_{sp} .$$

**Table 1:** Properties of base fluids and nanoparticles.

Material/Properties	ρ (kg/m^3^)	Cp (J/kg-K)	k (W/m–K)	μ (kg/m-s)
Water	997.1	4179	0.613	0.0010003
Fe_3_O_4_	5200	670	6	–

**Table 2:** Constant of Eq. (7).

Nanoparticle shape	**λ**
Spherical	1
Platelet	0.52
Cylindrical	0.62
Brick	0.81

As given in Table 1, for modeling the nanofluids, base fluid (water) and NPs (Fe_3_O_4_) in different shapes (brick, cylindrical, platelet and spherical) were considered. Viscosity of Fe_3_O_4_ was described by using the Brinkman Eq.^50^:

5 $$\mu_{na} = \frac{{\mu_{bf} }}{{\left( {1 - \emptyset } \right)^{2.5} }}.$$

The electrical conductivity ratio $\left( {\frac{{\sigma_{na} }}{{\sigma_{bf} }}} \right)$ is defined as;

6 $$\frac{{\sigma_{na} }}{{\sigma_{bf} }} = 1 + \frac{{3\emptyset \left( {\frac{{\sigma_{sp} }}{{\sigma_{bf} }} - 1} \right)}}{{\left( {\frac{{\sigma_{sp} }}{{\sigma_{bf} }} + 2} \right) - \left( {\frac{{\sigma_{sp} }}{{\sigma_{bf} }} - 1} \right)\emptyset }}.$$

The Hamilton equation is used to calculate the nanofluid thermal conductivity as;

7 $$\frac{{k_{na} }}{{k_{bf} }} = \frac{{k_{sp} + \left( {m - 1} \right)k_{bf} - \left( {m - 1} \right)\emptyset \left( {k_{bf} - k_{sp} } \right)}}{{k_{sp} + \left( {m - 1} \right)k_{bf} + \emptyset (k_{bf} - k_{sp)} }},$$

with m = 3 for spherical NPs. For other shapes, m is calculated from m = 3/λ as given in Table 2. The thermal properties of the computational domain are assumed to be constant unless the density, where the density division obedience to the Boussinesq approximation criteria. The dimensionless governing equations of the system are as follows:

A- For nanofluid domain;

Continuity equation

8 $$\frac{\partial U}{{\partial X}} + \frac{\partial V}{{\partial Y}} = 0$$

X-component momentum eqtion

9 $$\begin{aligned} & \left( {\frac{{\rho_{na} }}{{\rho_{bf} }}} \right) \left( {U \frac{\partial U}{{\partial X}} + V\frac{\partial U}{{\partial Y}}} \right) \\ & = - \left( {\frac{{\rho_{na} }}{{\rho_{bf} }}} \right)\frac{\partial P}{{\partial X}} + \frac{{\mu_{na} }}{{\mu_{bf} }}\left( {\frac{{\partial^{2} U}}{{\partial X^{2} }} + \frac{{\partial^{2} U}}{{\partial Y^{2} }}} \right) \\ & \quad + Ha^{2} \left( {\frac{{\rho_{na} }}{{\rho_{bf} }}} \right)\left( {\frac{{\sigma_{na} }}{{\sigma_{bf} }}} \right)\left( {Vsin\gamma cos\gamma - Usin^{2} \gamma } \right) \\ \end{aligned}$$

Y-component momentum equation

10 $$\begin{aligned} & \left( {\frac{{\rho_{na} }}{{\rho_{bf} }}} \right)\left( {U\frac{\partial V}{{\partial X}} + V\frac{\partial V}{{\partial Y}}} \right) = - \left( {\frac{{\rho_{na} }}{{\rho_{bf} }}} \right)\frac{\partial P}{{\partial Y}} + \frac{{\mu_{na} }}{{\mu_{bf} }}\left( {\frac{{\partial^{2} V}}{{\partial X^{2} }} + \frac{{\partial^{2} V}}{{\partial Y^{2} }}} \right) \\ & \quad + \left( {\frac{{\left( {\rho \beta } \right)_{na} }}{{\left( {\left( {\rho \beta } \right)_{bf} } \right)}}} \right)RaPr\theta_{na} + Ha^{2} \left( {\frac{{\rho_{na} }}{{\rho_{bf} }}} \right)\left( {\frac{{\sigma_{na} }}{{\sigma_{bf} }}} \right)\left( {Usin\gamma cos\gamma - Vcos^{2} \gamma } \right) \\ \end{aligned}$$

Energy equation

11 $$U\frac{{\partial \theta_{na} }}{\partial X} + V\frac{{\partial \theta_{na} }}{\partial Y} = \frac{{\alpha_{na} }}{{\alpha_{bf} }}\left( {\frac{{\partial^{2} \theta_{na} }}{{\partial X^{2} }} + \frac{{\partial^{2} \theta_{na} }}{{\partial Y^{2} }}} \right)$$

B- For a conductive hot rotating cylinder domain;
  12 $$\frac{{\partial^{2} \theta_{so} }}{{\partial X^{2} }} + \frac{{\partial^{2} \theta_{so} }}{\partial Y} = 0$$

Dimensionless parameters involved in the present study are:

13 $$\begin{aligned} X, X_{o} , Y,Y_{o} & = \frac{{x,x_{o} ,y,y_{o} }}{L}; U, V = \frac{{\left( {u,v} \right)L}}{{\nu_{bf} }}; \theta_{na} = \frac{{\left( {T_{na} - T_{c} } \right)k_{bf} }}{{q^{\prime \prime } L}}; \theta_{so} = \frac{{\left( {T_{so} - T_{c} } \right)k_{bf} }}{{q^{\prime \prime } L}}; \\ R & = \frac{r}{L};Pr = \frac{{\nu_{bf} }}{{\alpha_{bf} }};Ra = \frac{{g\beta_{bf} q^{\prime \prime } L^{4} }}{{k_{bf} \nu_{bf} \alpha_{bf} }};P = \frac{{pL^{2} }}{{\rho_{bf} \alpha_{bf}^{2} }}; \Omega = \frac{{\omega L^{2} }}{{\alpha_{bf} }}; \Psi = \frac{\psi }{{\left( {\rho \alpha } \right)_{bf} }} \\ \end{aligned}$$

Dimensionless number for the case study in this work are:

14 $$\begin{aligned} & \left( {Rayleigh\,number} \right)Ra = \frac{{g\beta_{bf} q^{\prime \prime } L^{4} }}{{k_{bf} \nu_{bf} \alpha_{bf} }}, \left( {Prandtl\, number} \right)Pr \\ & \quad \quad \quad \quad = \frac{{\mu_{bf} }}{{\alpha_{bf} \rho_{bf} }}, \left( {Grashof \,number} \right)Gr = \frac{Ra}{{Pr}}, \left( {Hartmann \,number} \right)Ha \\ & \quad \quad \quad \quad = BL\sqrt {\frac{{\sigma_{na} }}{{\rho_{na} \nu_{bf} }}} , \,\,\,\,\,\,dimensionless \,angular \,velocity\,\,\,\, {\Omega } = \frac{{\omega L^{2} }}{{\alpha_{bf} }} \\ \end{aligned}$$

Dynamic and thermal boundary conditions in this work are:

1. On the adiabatic horizontal top walls
  15 $$U = V = 0; \frac{{\partial \theta_{na} }}{\partial Y} = 0$$
2. On bottom horizontal wall and two vertical left and right walls
  16 $$U = V = 0; T = T_{c} \to \theta_{na} = 0$$
3. Linear velocity in X and Y-direction for rotating cylinder can be described as;
  17 $$U = - \Omega \left( {Y - Y_{o} } \right);\,\,\,{\text{and}}\,\,\, V = \Omega \left( {X - X_{o} } \right).$$

The heat-transfer equilibrium between the rotating cylinder and square enclosure;

18a $$\theta_{na} = \theta_{so}$$

18b $$k_{c} \left( {U\frac{{\partial \theta_{na} }}{\partial X} + V\frac{{\partial \theta_{na} }}{\partial Y}} \right) = k_{r} \left( {\left[ {\frac{{\partial^{2} \theta_{so} }}{{\partial X^{2} }} + \frac{{\partial^{2} \theta_{so} }}{{\partial Y^{2} }}} \right]} \right)$$

where $k_{c} ; k_{r}$ refers to

19 $$k_{c} = \frac{{\left( {\rho c_{p} } \right)_{so} }}{{\left( {\rho c_{p} } \right)_{na} }};\quad and\quad k_{r} = \frac{{k_{so} }}{{k_{na} }}.$$

The energy-conservation between the rotating cylinder boundary and porous media, in the normal direction to the cylinder surface, causes the energy balance as;

20 $$k_{na} \left( {\frac{{\partial \theta_{na} }}{\partial n}} \right) = k_{so} \left( {\frac{{\partial \theta_{so} }}{\partial n}} \right)\, \to \,\left( {\frac{{\partial \theta_{na} }}{\partial n}} \right) = k_{r} \left( {\frac{{\partial \theta_{so} }}{\partial n}} \right).$$

When the conductive rotating cylinder is considered a constant heat-flux $(q^{\prime \prime } )$, the energy balance will be:

21 $$q_{cond.} = q^{\prime \prime } \, \to \, k_{so} \frac{{\partial \theta_{so} }}{\partial n} = q^{\prime \prime } \, \to \,\frac{{\partial \theta_{so} }}{\partial n} = \frac{{q^{\prime \prime } }}{{k_{na} }}.$$

The flow structure can be defined by streamlines contours as follows;

22a $$\frac{\partial \Psi }{{\partial X}} = - V; \frac{\partial \Psi }{{\partial Y}} = U,\quad and$$

22b $$\frac{{\partial^{2} {\Psi }}}{{\partial X^{2} }} + \frac{{\partial^{2} {\Psi }}}{{\partial Y^{2} }} = \left( {\frac{\partial U}{{\partial Y}} - \frac{\partial V}{{\partial X}}} \right).$$

The local Nusselt number was computed along the arc of contact between the solid cylinder (conduction) and nanofluid (convection) in Eq.^51^,

23 $$Nu_{loc} = \left( {\frac{{k_{na} }}{{k_{bf} }}} \right)\frac{\partial \theta }{{\partial \xi }}.$$

Finally, average Nusselt number was calculated from integration of the local Nusselt number along the same arc as per Eq.^51^;

24 $$Nu_{ave} = \frac{1}{{l_{arc} }}\mathop \smallint \limits_{0}^{{l_{arc} }} Nu_{loc} d\xi .$$

## Numerical method and verification

The governing equations in the dimensionless form Eqs. (9) – (12) are solved numerically using Galerkin FEM to find the stream function and dimensionless temperature scattering inside the porous cavity in presence of magnetic field and rotating cylinder. Natural, mixed and forced convection are solved by FEM, which gives more accurate results, decreasing the requirement of computer storage and time of solution^36^. Penalty formulation is used to eliminate the pressure term (P) in the momentum equations with a Penalty parameter ($\vartheta$) using Eq.;

25 $$P = - \vartheta \left( {\frac{\partial U}{{\partial X}} + \frac{\partial V}{{\partial Y}}} \right).$$

After inserting above equation in the momentum Eqs. (9), (10);

26 $$\begin{aligned} & \left( {\frac{{\rho_{na} }}{{\rho_{bf} }}} \right)\left( { U \frac{\partial U}{{\partial X}} + V\frac{\partial U}{{\partial Y}}} \right) = \left( {\frac{{\rho_{na} }}{{\rho_{bf} }}} \right)\frac{\partial \vartheta }{{\partial X}}\left( {\frac{\partial U}{{\partial X}} + \frac{\partial V}{{\partial Y}}} \right) + \frac{{\mu_{na} }}{{\mu_{bf} }}\left( {\frac{{\partial^{2} U}}{{\partial X^{2} }} + \frac{{\partial^{2} U}}{{\partial Y^{2} }}} \right) \\ & \quad + Ha^{2} \left( {\frac{{\rho_{na} }}{{\rho_{bf} }}} \right)\left( {\frac{{\sigma_{na} }}{{\sigma_{bf} }}} \right)\left( {Vsin\gamma cos\gamma - Usin^{2} \gamma } \right),\quad and \\ \end{aligned}$$

27 $$\begin{aligned} & \left( {\frac{{\rho_{na} }}{{\rho_{bf} }}} \right)\left( {U\frac{\partial V}{{\partial X}} + V\frac{\partial V}{{\partial Y}}} \right) = \left( {\frac{{\rho_{na} }}{{\rho_{bf} }}} \right)\frac{\partial \vartheta }{{\partial Y}}\left( {\frac{\partial U}{{\partial X}} + \frac{\partial V}{{\partial Y}}} \right) + \frac{{\mu_{na} }}{{\mu_{bf} }}\left( {\frac{{\partial^{2} V}}{{\partial X^{2} }} + \frac{{\partial^{2} V}}{{\partial Y^{2} }}} \right) \\ & \quad + \left( {\frac{{(\rho \beta )_{na} }}{{\left( {\rho \beta } \right)_{bf} }}} \right)\frac{Ra}{{\Pr }}\theta_{na} + Ha^{2} \left( {\frac{{\rho_{na} }}{{\rho_{bf} }}} \right)\left( {\frac{{\sigma_{na} }}{{\sigma_{bf} }}} \right)\left( {Usin\gamma cos\gamma - Vcos^{2} \gamma } \right) \\ \end{aligned}$$

**Figure 2:**
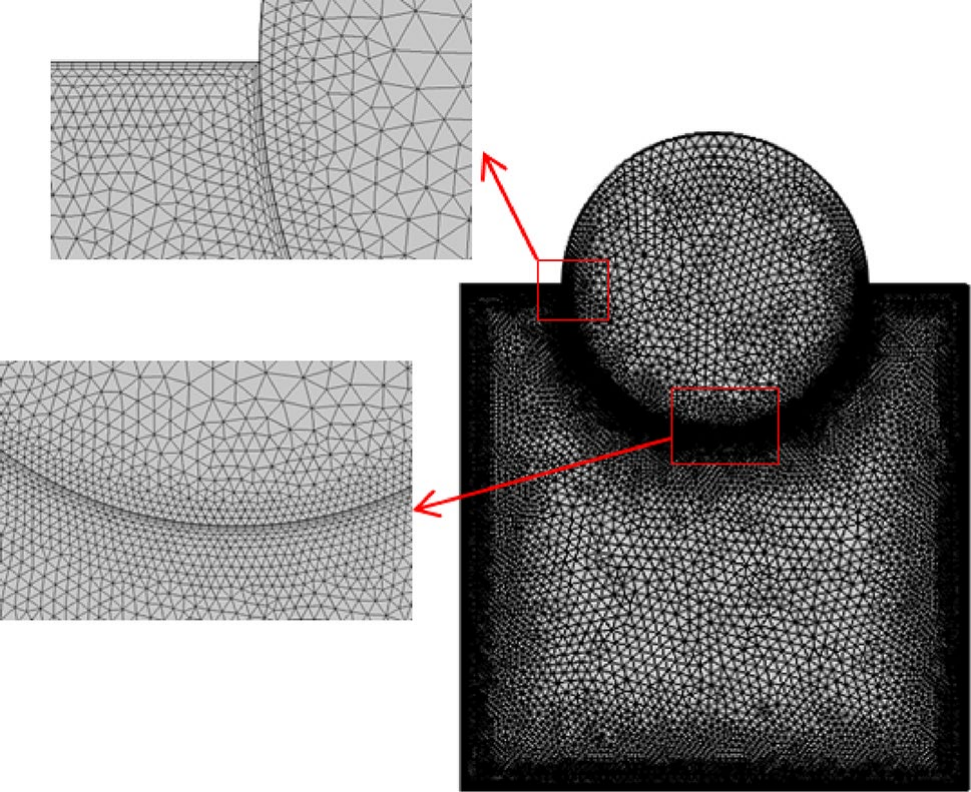
Mesh distribution of the physical domain.

A triangular shape of elements in Fig. 2 was selected, and the integration of momentum equations utilizing weak formulation (weighted-integral) were depended over the computational domain. Lagrange finite elements with polynomial degree are utilized to solve a set of partial differential equations and used to discretize the X and Y direction velocities, temperature and pressure in the domain. By applying initiation functions, the variables within the domain were separated into non-flapping zones. After replacing the variables to the dimensions governing relations, residuals will be produced and must be resolved to emphasize equal to zero up the computational domain as:

28 $$\int\limits_{\tau } {WDd\tau } = 0$$

where W represents the weight function in Galerkin method, and is subtituted from the equivalent arranged of functions named trial functions. Velocity, temperature and stream function variables were estimated using the function of interpolation as follows.

29 $$U \approx \mathop \sum \limits_{i = 1}^{N} U_{i} \Gamma_{i} \left( {X, Y} \right); \,\,\,\,V \approx \mathop \sum \limits_{i = 1}^{N} V_{i} \Gamma_{i} \left( {X, Y} \right);\,\,\,\,\theta \approx \mathop \sum \limits_{i = 1}^{N} \theta_{i} \Gamma_{i} \left( {X, Y} \right);\,\,\,\,\Psi \approx \mathop \sum \limits_{i = 1}^{N} \Psi_{i} \Gamma_{i} \left( {X, Y} \right).$$

By production for each node of the component or element, a nonlinear residual equation will be obtained, which is calculated by Newton–Raphson scheme. The iteration of the current problem is expected to reach convergence results on an error ≤ 10^–5^ for each variable.

**Figure 3:**
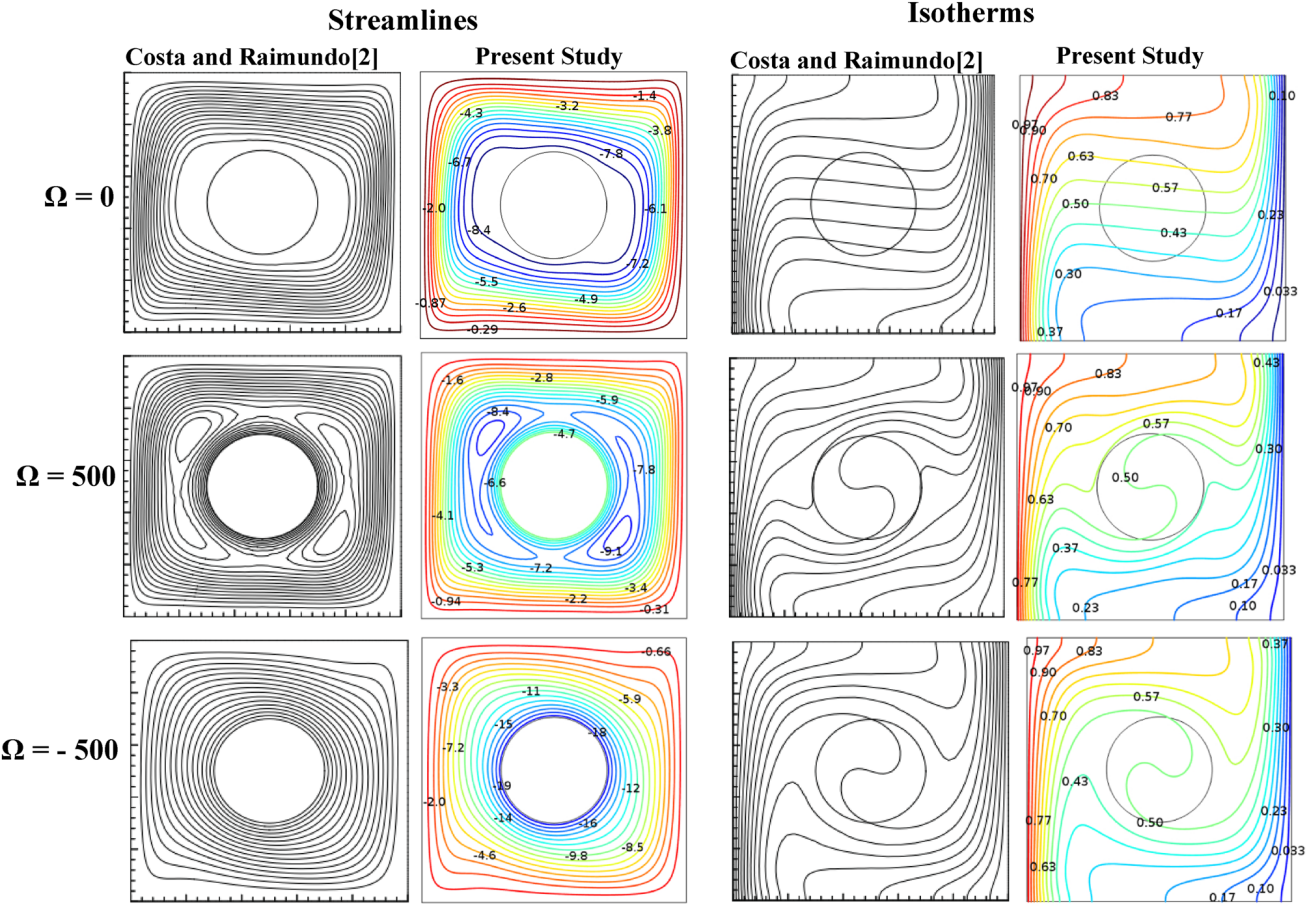
Comparison of streamlines and isotherms between Costa and Raimundo^2^ and present study at different angular rotational velocities Ω (R = 0.4 H, Rc = 1, and R_k_ = 1).

**Figure 4:**
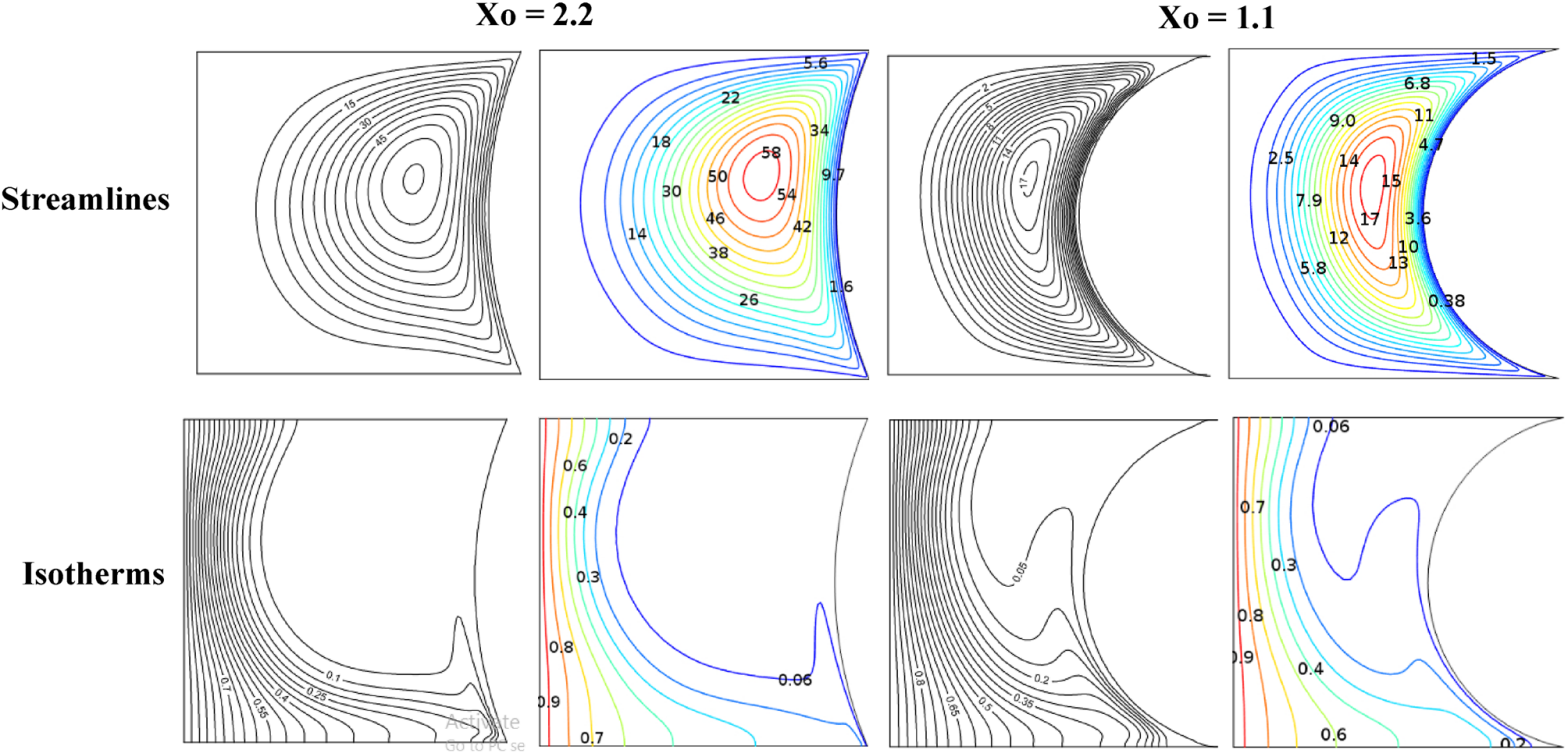
Streamlines (upper row) and isotherms (lower row) for Ra = 10^4^ and ω = 500.

Non-homogenous distribution, triangular mesh element is used to grid both domains of porous medium and nanofluid. Different types of mesh sizes, boundary elements, number of elements, time elapsed and error were considered in Table 3 to check the grid sensitivity of this model. An average Nusselt number for the hot surface is depicted for the results analysis. In a case study with the conditions (Ra = 10^6^, Ω = 500, *ϕ* = 0.05, λ = 0.52, Ha = 60, and γ = 45°), a minimum error ~ 0.01% at extremely fine mesh with 20,564 number of elements and boundary elements equal to 714 and elapsed time of 29 s. Figure 2. represents the mesh distribution of the physical domain with an enlarge view of mesh generation in the contact area between the solid cylinder and nanofluid. The created mesh near the boundaries was very fine to sense physical changes and obtain accurate results. To find the accuracy of the numerical method of the current computational software, fluid flow structure is presented by streamlines contour and heat-transfer is analyzed by isotherms contour and average Nusselt number. The outcomes were compared with previous numerical studies performed by Costa and Raimundo^2^ and Ismael^30^ as depicted in Figs. 3 and 4, Tables 4 and 5, respectively. It is articulate that a very good agreement between the results of the numerical approach is observed, demonstrating validity of the present numerical code that it is reliable and suitable for next studies in general.

**Table 3:** Grid testing for *average Nussult number* on hot surface (Ra = 10^6^, λ = 0.52, ϕ = 0.05, Ha = 60, Ω = 500, and γ = 45°).

Grid size	Number of elements	Boundary elements	Average Nussult number Nu	t	Error (%)
G1	1167	115	1.2236	8	–
G2	1677	134	1.2209	8	− 0.27
G3	2640	176	1.2222	9	0.13
G4	7673	371	1.2404	14	0.18
G5	20,564	714	1.2479	29	0.75
G6	31,186	732	1.2478	37	− 0.01

**Table 4:** Comparison of the average Nusselt number between the present study and Costa and Raimundo^2^, the result for different angular rotational velocities Ω (R = 0.4 H, R_c_ = 1, and R_k_ = 1).

Ω	Average Nusselt number at the hot wall		Error (%)
	Present study	Costa and Raimundo^2^	
0	4.52	4.52	0
500	4.4117	4.41	− 0.038
− 500	4.276	4.27	− 0.0014

**Table 5:** Variation of average Nusselt number with ω along the left hot wall for x_o_ = 1.3 and Ra = 10^3^.

ω	Ismael^30^		Present work
	Nu_ave_	Error (%)	
− 1000	5.95	5.85	1.68
− 600	4.8	4.74	1.25
− 200	3.33	3.27	1.8
0	1.35	1.35	0
200	2.92	2.93	− 0.34
600	4.55	4.55	0
1000	5.7	5.68	0.35

## Results and discussion

**Figure 5:**
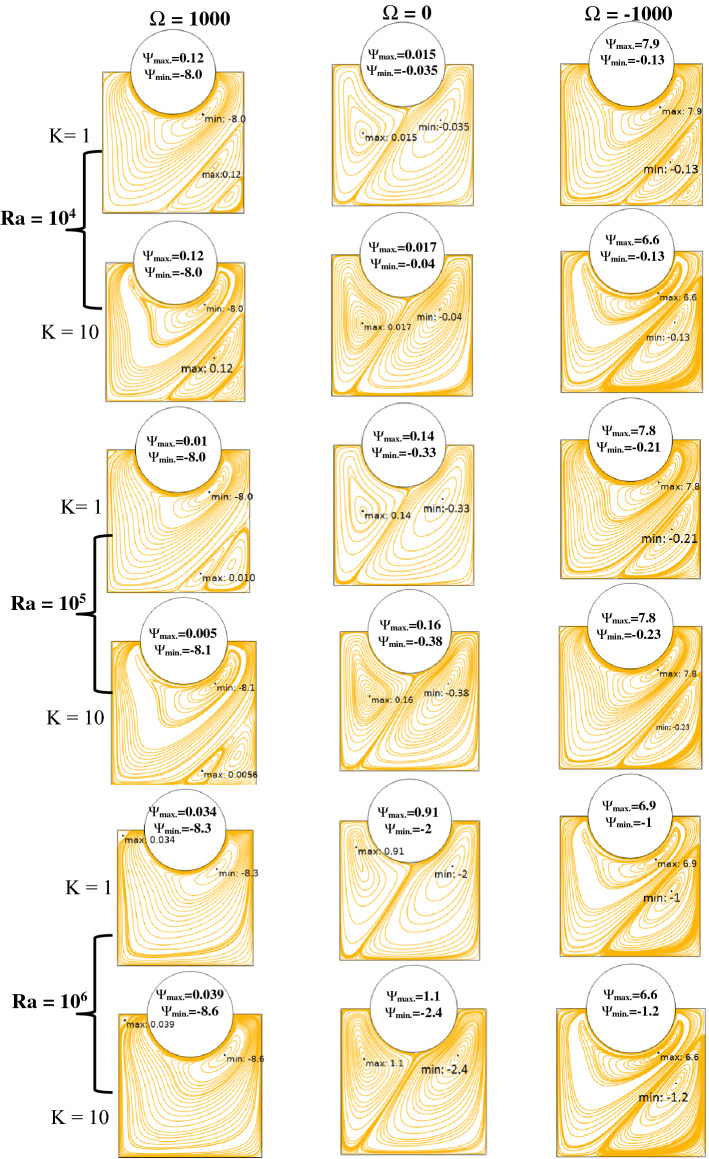
Streamlines for different Rayleigh numbers, dimensionless rotating angular velocities, and thermal conductivity ratios at Ha = 60, λ = 0.52, and ϕ = 0.05.

**Figure 6:**
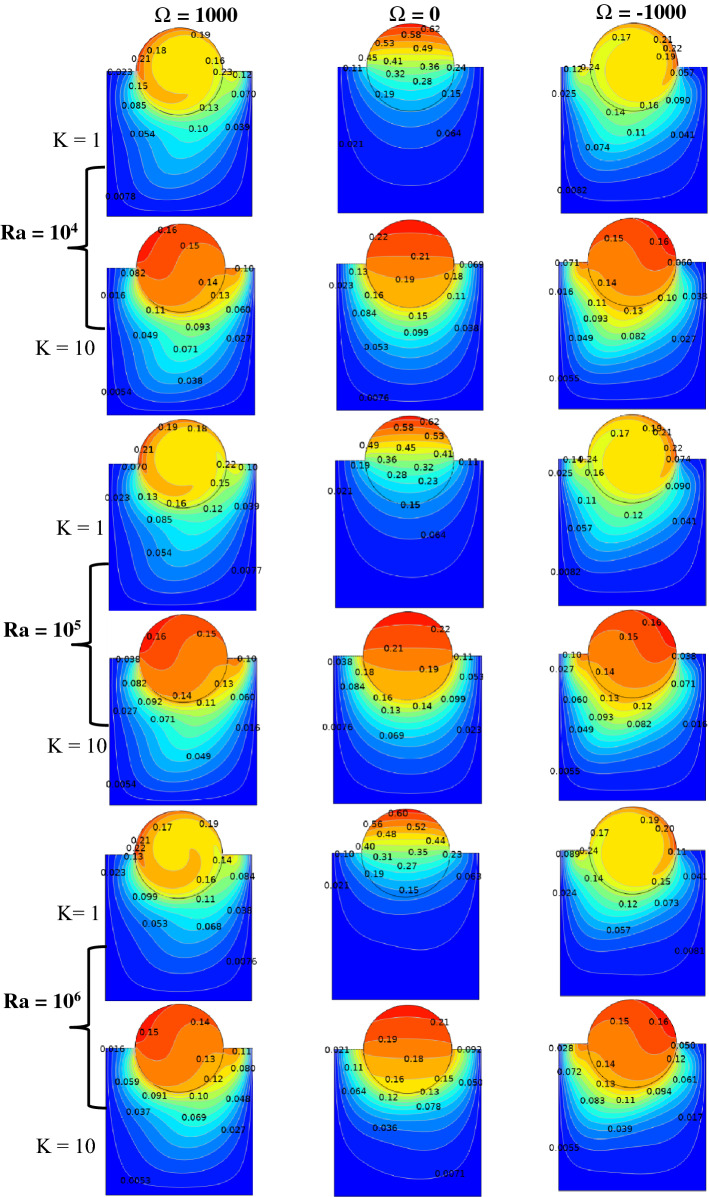
Isotherms for different Rayleigh numbers, dimensionless rotating angular velocities, and thermal conductivity ratios at Ha = 60, λ = 0.52, ϕ = 0.05, and γ = 45°.

The validity of applied Galerkin FEM is examined by comparing the results with the literature values^2,30^, as portrayed in Figs. 3, 4, and Tables 4, 5, respectively. A maximum difference between the Nusselt numbers in different angular velocities is 1.8%, i.e. an acceptable error in the numerical solution. Authors used Ra to illustrate the effect of buoyancy force on fluid structure and temperature distribution inside a square enclosure. Ra and Ω are used instead of Richardson number (Ri). Figures 5, 6 are depicted to find the effect of Ra, rotating angular velocity and thermal conductivity ratio at the same time for streamlines and isotherm lines, respectively. As seen in Fig. 5, when the angular velocity is zero, the separation line of streamlines is along the magnetic direction angle, γ = 45°, but when the cylinder rotates (clockwise or counterclockwise directions) it influences the vortexes and separation of two main vortexes moves to right or left sides, respectively. Also, this figure reveals that an increasing thermal conductivity ratio (especially in low Ra values) causes the streamlines more turbulent. Furthermore, an increasing Ra value makes a significant reduction of the maximum values of streamlines. A maximum value of the streamlines occurred on K = 1, Ω = -1000 and Ra = 10^4^. It means it reduces on larger thermal conductivity ratio and Ra values. From Fig. 6, it can be concluded that an increasing Ra promotes the natural convection heat-transfer, so temperature values will be smaller on higher Ra values. Also, in large thermal conductivity rations (K = 10), cylinder has smaller temperature values due to more heat-transfer to nanofluid. As seen in this figure, temperature contours on the cylinder is stationary is nearly symmetric, but by rotating the cylinder, temperature contours will change to asymmetric shape and more temperature values occur in the sides which it revolves. A minimum cylinder temperature and so more heat-transfer to nanofluids occured when K = 10, Ra = 10^6^ and Ω = -1 000 or 1000. This confirms that rotation of cylinder makes faster heat-transfer from cylinder to nanofluid as well as larger thermal conductivity ratio.

**Figure 7:**
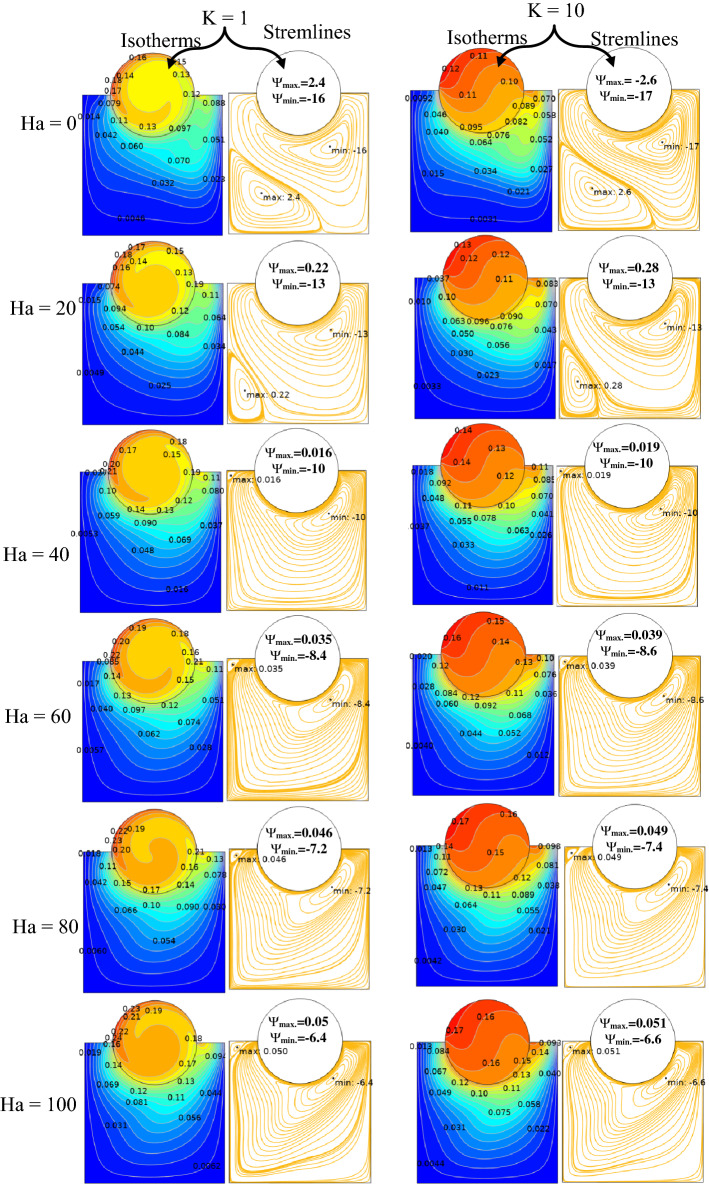
Isotherms and streamlines contours for different Hartmann numbers and thermal conductivity ratios at Ra = 10^6^, λ = 0.52, and ϕ = 0.05.

**Figure 8:**
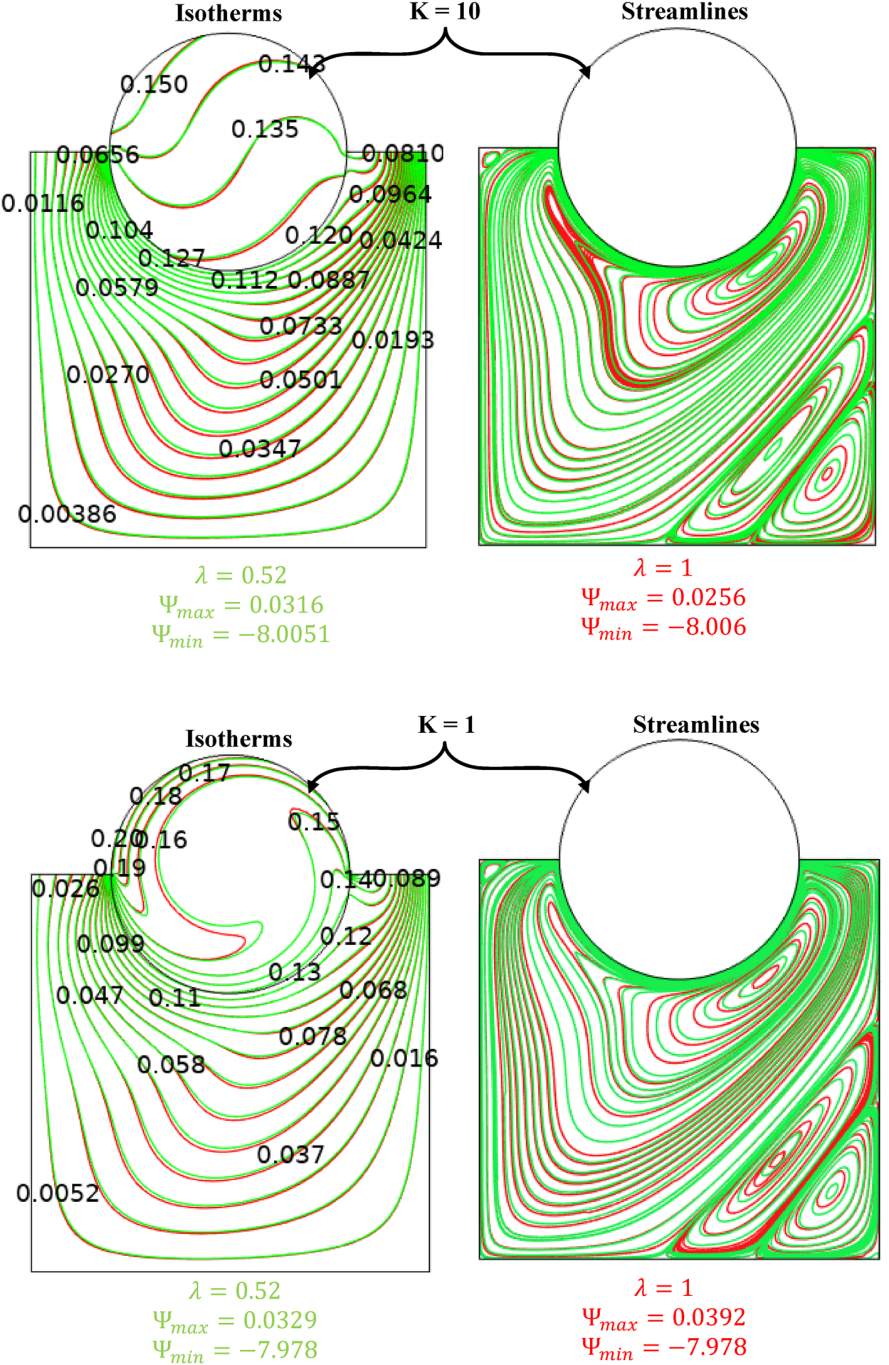
Isotherms and streamlines contours for different NPs shapes and thermal conductivity ratios at Ra = 10^5^, Ha = 60, and ϕ = 0.05.

**Figure 9:**
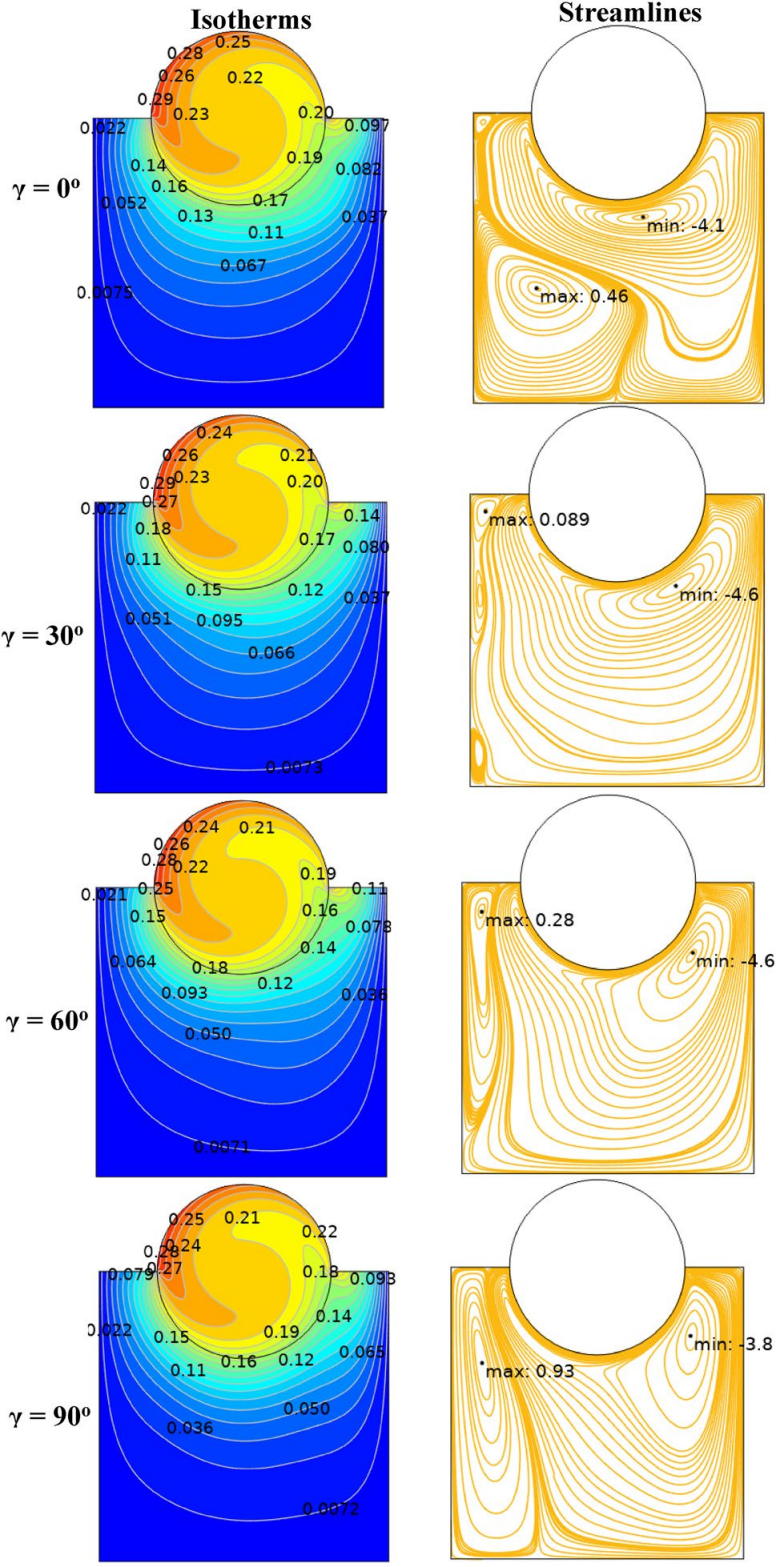
Isotherms and streamlines contours for different inclination angles, with Ra = 10^6^, Ha = 60, ϕ = 0.05, K = 1, Ω = 500, and λ = 0.52.

**Figure 10:**
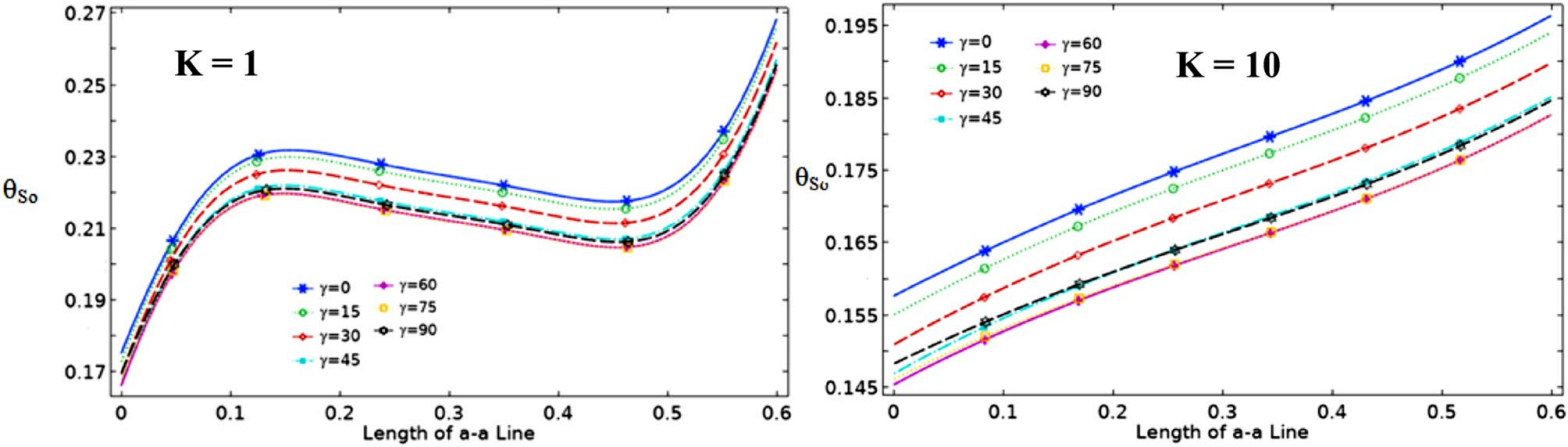
Dimensionless temperature along line a-a for different angles of magnetic field at Ra = 10^6^, Ha = 60, ϕ = 0.05, Ω = 500, and λ = 0.52.

**Figure 11:**
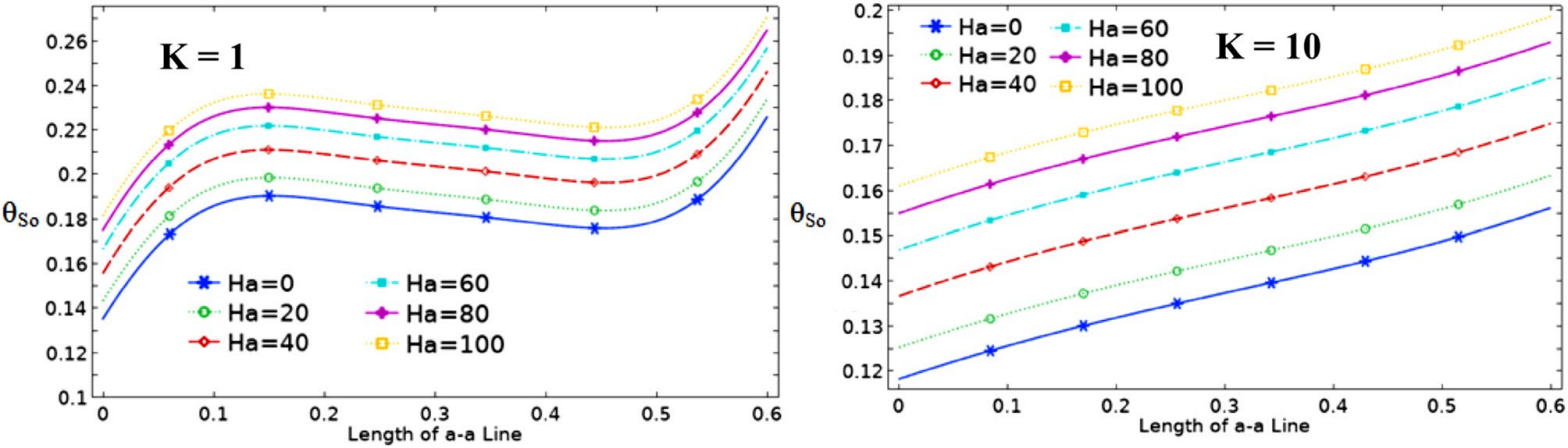
Dimensionless temperature along line a-a for different Hartmann numbers at Ra = 10^6^, ϕ = 0.05, Ω = 500, and λ = 0.52.

**Figure 12:**
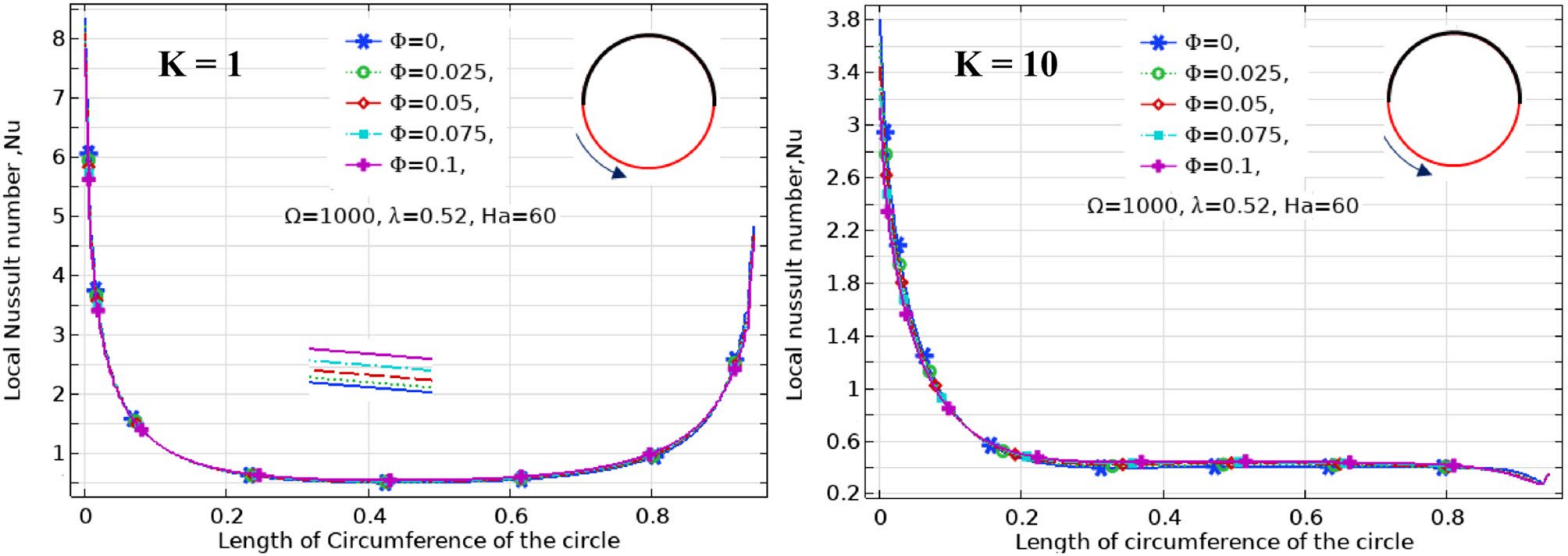
Local Nusselt number along contact arc for different NPs volume fractions at Ra = 10^6^, Ha = 60, Ω = 1000, and λ = 0.52.

**Figure 13:**
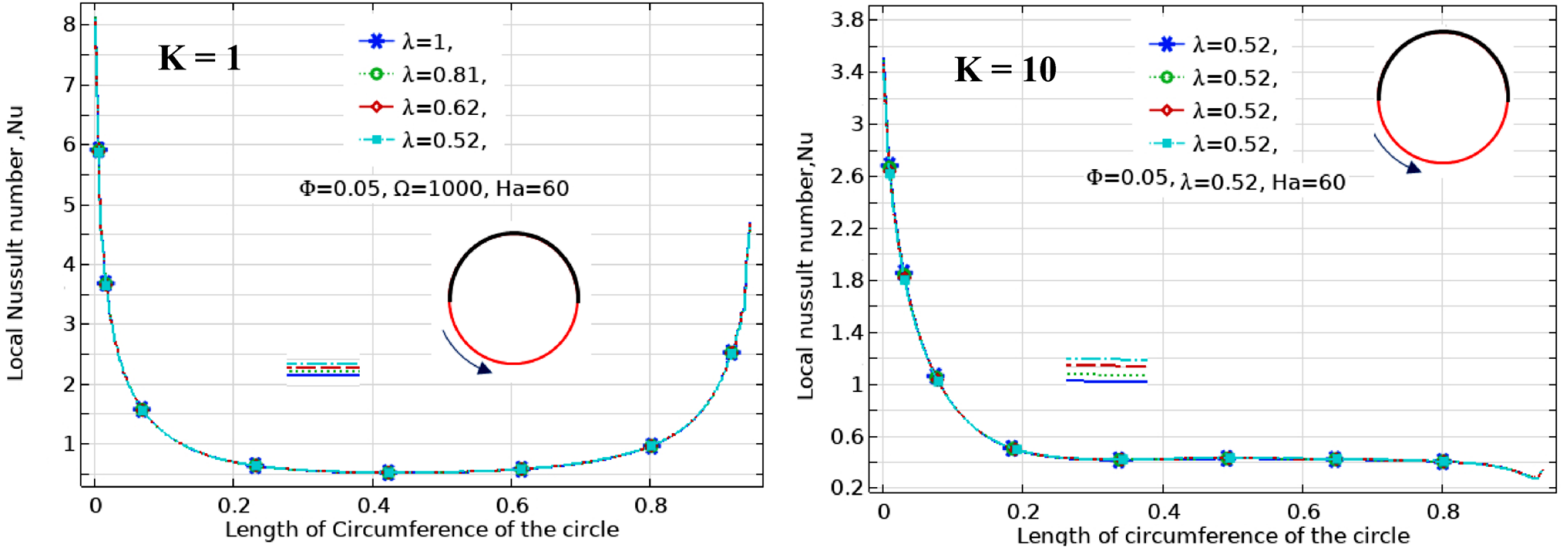
Local Nusselt number along contact arc for different NPs shapes at Ra = 10^6^, Ha = 60, Ω = 1000, and ϕ = 0.05.

**Figure 14:**
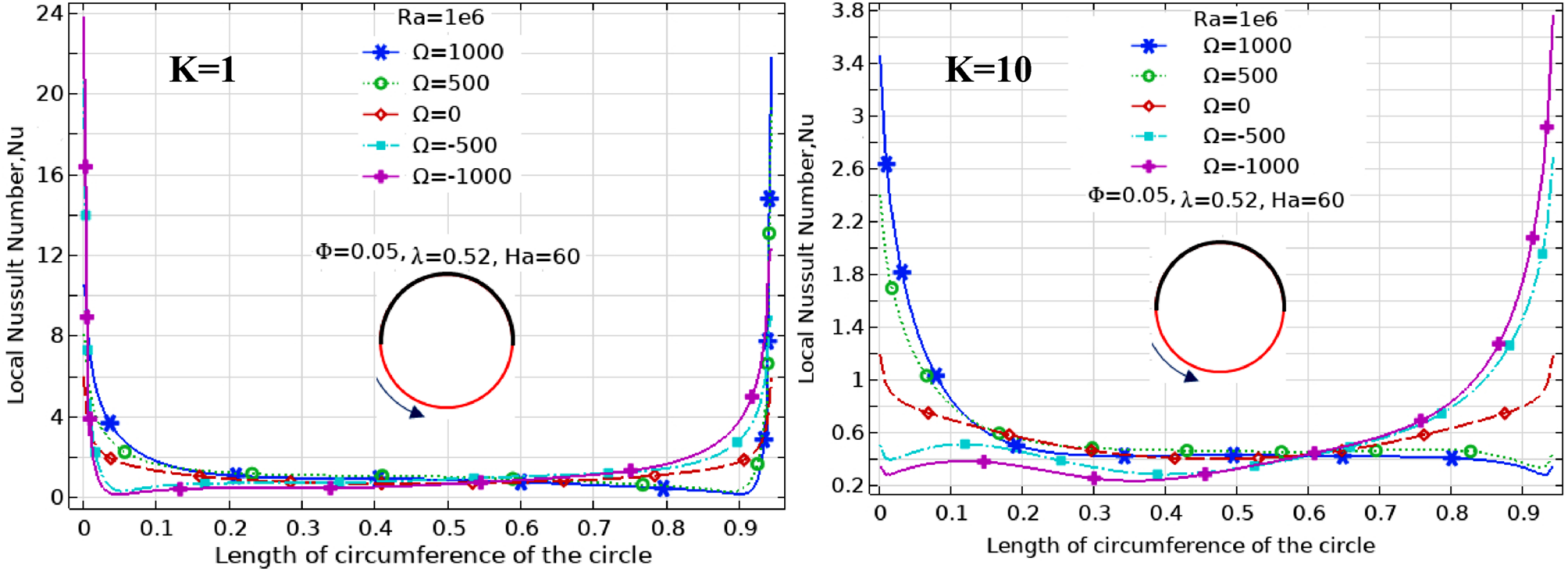
Local Nusselt number along contact arc for different rotationl speeds at Ra = 10^6^, Ha = 60, λ = 0.52, and ϕ = 0.05.

The effect of Ha on the temperature and streamlines is presented in Fig. 7, with Ra = 10^6^, λ = 0.52, and ϕ = 0.05. Larger the Ha larger temperature values in both nanofluid and cylinder temperatures, i.e. less heat-transfer to boundaries due to a magnetic effect on the NPs motion and heat-transfer, consequently. It confirms an increased Ha significantly reduces the maximum value of streamline at low Ha = 0–20, but a larger Ha leads to raise maximum streamline values. All Figs. 3–7 are presented at λ = 0.52 of platelet NPs. Figure 8 compares the results with spherical shaped NPs, confirming spherical NPs have greater values of temperature, i.e. lower heat-transfer to boundaries. Also, the maximum values of streamlines for the spherical NPs (on K = 1) is larger than platelet NPs, while a reversed treatment is observed at K = 10. The last contour depicted here is presented in Fig. 9 to find the effect of magnetic inclination angle on the temperatures and streamlines when Ra = 10^6^, Ha = 60, ϕ = 0.05, K = 1, Ω = 500, and λ = 0.52 . Although the inclination angle has no significant effect on the isotherm lines, it duly tunes the streamlines pattern. A maximum temperature value for the cylinder at all inclination angles is ~ 0.29, while the maximum value of streamline varies from 0.46 (γ = 0) to 0.089 (γ = 30). To have a better perception of K and γ effects on the temperatures, Fig. 10 is depicted on the a-a line of cylinder diameter as shown in Fig. 1. This figure shows that an increasing magnetic angle reduces the temperature along the defined line as well as the thermal conductivity ratio. So, both parameters favor the heat-transfer. Also, this figure confirms that, at K = 10, the temperature along a-a line over the cylinder is more linear than at K = 1, and maximum values at K = 1 are greater than at K = 10. Figure 11 reveals that Ha has a solely different behavior of it leads to raise the temperatures along the (a-a) line, i.e. lower heat-transfer to nanofluid and so smaller Nusselt numbers. Local Nusselt numbers are displayed in Figs. 12–14. Figure 12 reveals that an increasing NPs fraction promotes local Nusselt number, i.e. more heat-transfer on greater thermal conductivity of nanofluid. Effect of NPs shape on the local Nusselt number is presented in Fig. 13. At K = 1, platelet NPs have larger Nusselt numbers among the tested shapes, while at K = 10, spherical NPs had their maximum values. Figure 14 displys the effect of rotational speed on the local Nusselt numbers. As cylinder rotation promotes heat-transfer, so the rotational speed leads to raise those on more turbulent fluid.

**Figure 15:**
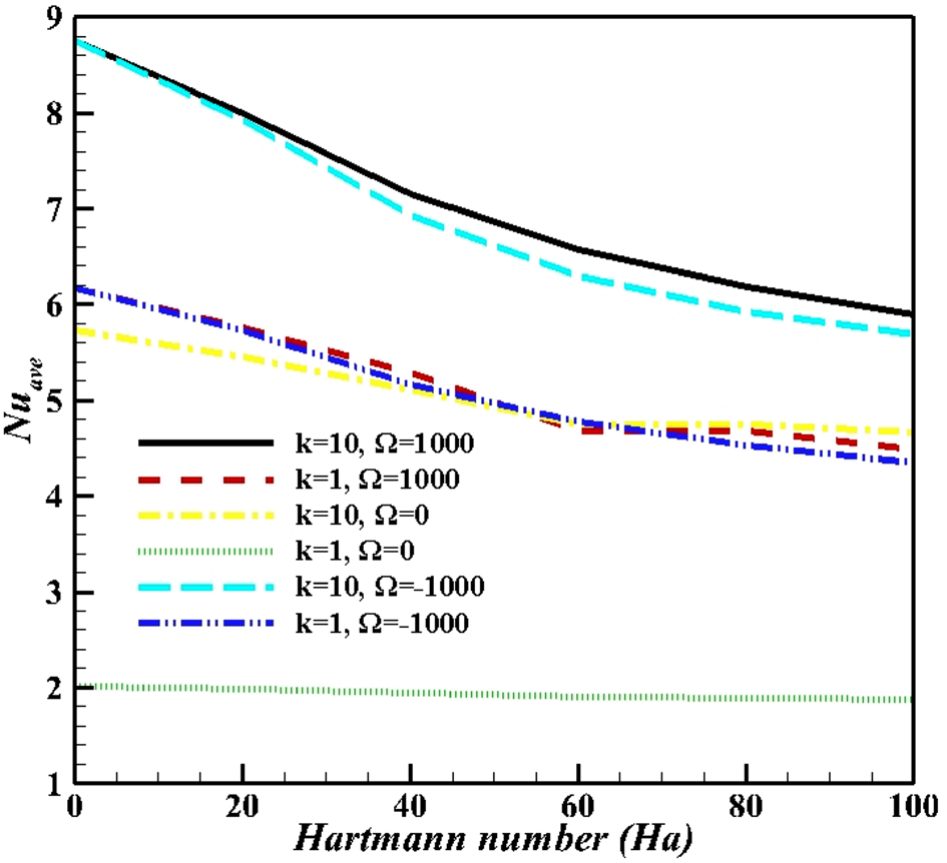
Average Nusselt number for different Hartman numbers at Ra = 10^6^, ϕ = 0.05, and λ = 0.52.

**Figure 16:**
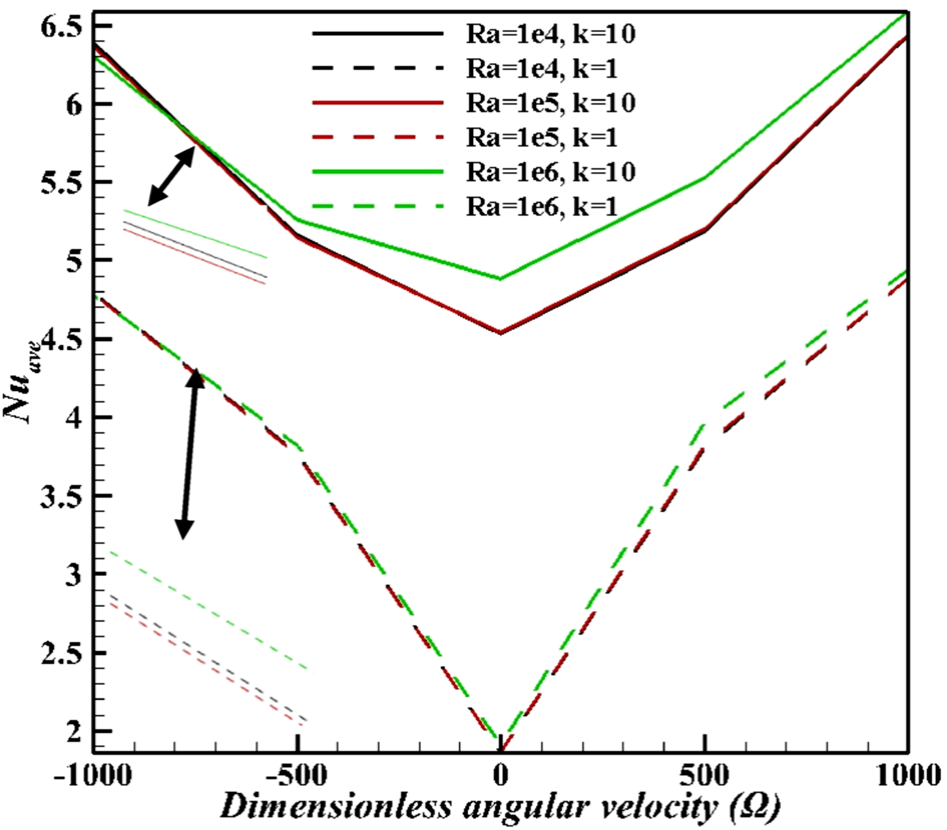
Average Nusselt number for different Raleigh numbers, dimensionless angular velocities, and thermal conductivity ratios at Ha = 60, ϕ = 0.05, and λ = 0.52.

**Figure 17:**
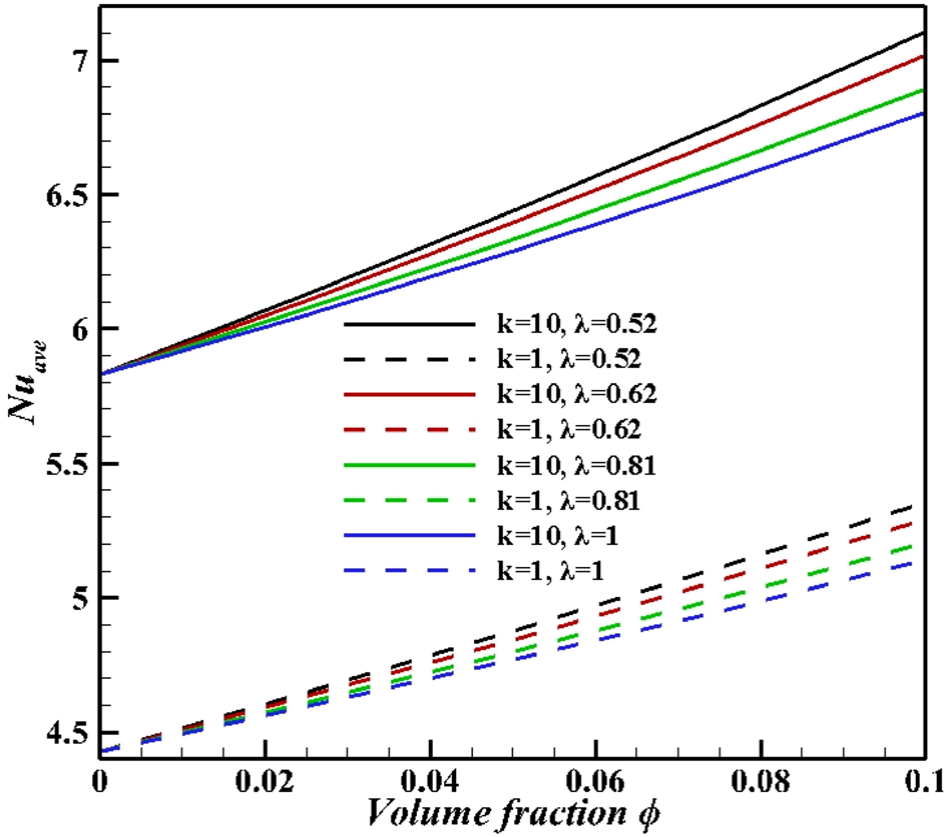
Average Nusselt number for different volume fractions, NPs shapes, thermal conductivity ratios at Ra = 10^5^, Ha = 60, ϕ = 0.05, and Ω = 1000.

**Figure 18:**
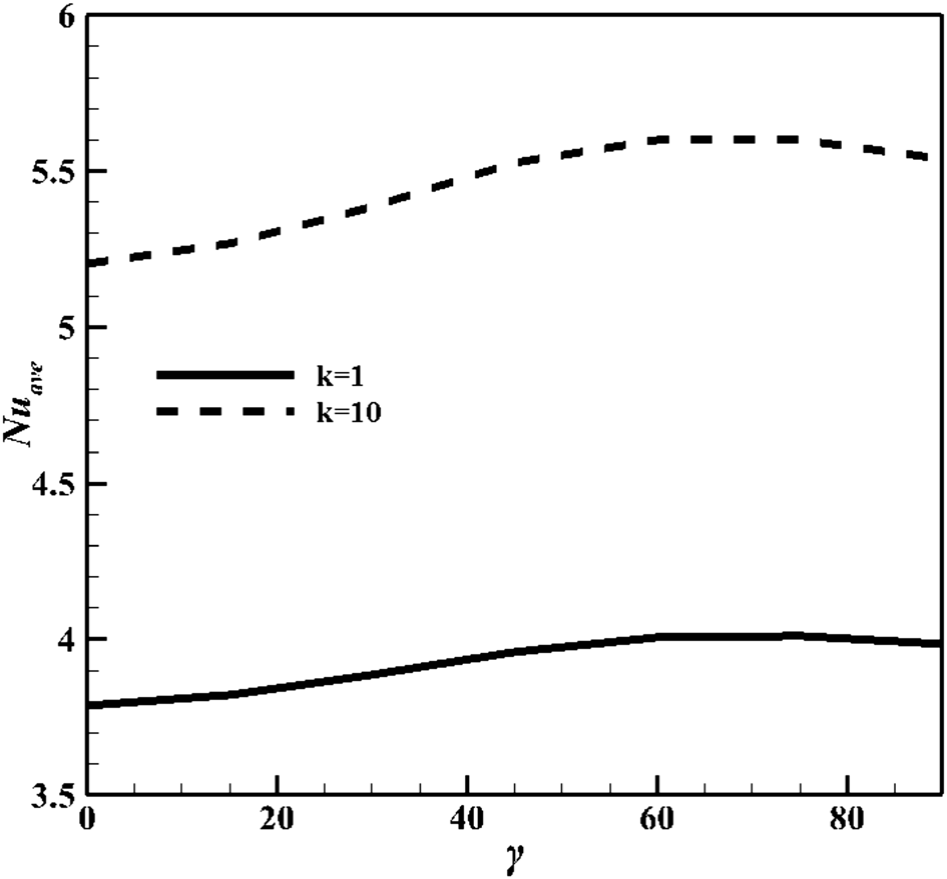
Average Nusselt number for different inclination angles of magnetic field, thermal conductivity ratios at Ra = 10^6^, Ha = 60, ϕ = 0.05, Ω = 500, and λ = 0.52.

The results of average Nusselt number defined in the above half-circle section of the cylinder are presented in Figs. 15–18. As illustared in Fig. 15, an increasing thermal conductivity ratio promotes the heat-transfer to nanofluid, with increasing lower arc Nusselt number. Also, raising rotational speed will enhance the average Nusselt number. Further, rotating the cylinder (in both directions) makes a greater Nusselt number due to more vortex generation and enhanced heat-transfer. An applied magnetic field suppressed average Nusselt numbers in account of induced magnetic force on Fe_3_O_4_ NPs. An increasing Ra value in Fig. 16, over dominant natural convection mechanism, favors average Nusselt number. Symmetrical shape of graph is due to minimum Nusselt number for zero angular velocity, while that for 1000 and -1000 it is at maximum values. The effects of NPs vaolume fraction and shape on the average Nusselt number is presented in Fig. 17. NPs promote Nusselt numbers due to larger thermal conductivity of nanofluids. Platelet NPs (λ = 0.52) have the maximum average Nusselt number, while the spherical NPs (λ = 1) have the minimum values for both K = 1 and 10. The order of NPs shapes from the larger Nusselt numbers is platelet > cylindrical > brick > spherical. Higher thermal conductivity (See Eq. 7) for different shapes of nanoparticles as well as the greater surface/volume ratio of nanoparticles are the main reasons of this treatment. Finally, results in Fig. 18 confirm the results in Fig. 15. As mentioned in Fig. 15, increasing inclination magnetic angle and thermal conductivity ratio promote the Nusselt numbers, but the effect of latter is more significant.

## Conclusions

Conjugate mixed convection of rotating conductive cylinder inside the nanofluid-filled cavity has been studied numerically using the Galerkin weighted residual finite element method. This phenomenon can be seen in several places, including asphalt paving when paving roads. Or parts of heavy machinery and equipment represented by rotary bar bears, ball bearing and different metal sheet rolling plants. The cylinder has exposed to heat-flux under the magnetic field at varied inclination angles. The influence of Rayleigh number, Hartmann number, NPs volume fraction, and shapes of NPs on the heat-transfer mechanism is explored, wherein an increasing Rayleigh number promotes the heat-transfer process as per the NPs volume fraction. In general, the Hartmann number has a different treatment. Platelet Fe_3_O_4_ NPs have the best heat-transfer on maximum Nusselt numbers. As the future study, authors propose to researchers to include different forms of cavities in other applications, for example a semicircular cavity with the presence of a half-submerged rotating cylinder, or shift the cylinder to one side and model the problem in three dimensional geometry. Also, considering the electrical field effect on other type of nanofluids may be useful for researchers.

## List of symbols

C_p_: Specific heat at constant pressure (KJ/kg.K)
g: Gravitational acceleration (m/s^2^)
k: Thermal conductivity (W/m.K)
L: Length & height of the cavity (m)
P: Dimensionless pressure
p: Pressure (Pa)
r: Radius (m)
Pr: Prandtle number (ν_f_/α_f_)
R: Non-dimensional radius of the solid cylinder
Ra: Rayleigh number $(g\beta_{bf} L^{3} \left( {T_{h} - T_{\infty } } \right)/\nu_{bf} \alpha_{bf} )$
T: Temperature (K)
T_c_: Temperature of the cold surface (K)
T_h_: Temperature of the hot surface (K)
Ha: Hartman number
qʺ: Heat flux (W/m^2^)
*N*: Number of nodes
Nu_loc_: Local Nusselt number
Nu_ave_: Average Nusselt number
U: Dimensionless velocity component in x-direction
u: Velocity component in x-direction (m/s)
V: Dimensionless velocity component in y-direction
v: Velocity component in y-direction (m/s)
X, Y: Dimensionless coordinate
x, y: Cartesian coordinates (m)
X_0_, Y_0_: Dimensionless coordinate of the rotating cylinder
x_0_, y_0_: Cartesian coordinate in
k_r_: Thermal conductivity ratio
B: Magnetic strength (Tesla)

## Greek symbols

α: Thermal diffusivity (m^2^/s)
θ: Dimensionless temperature
${\uppsi }$: Dimensional stream function (m^2^/s)
${\Psi }$: Dimensionless stream function
μ: Dynamic viscosity (kg.s/m)
$$\xi$$: Rotation angle
γ: Magnetic field inclination angle
$${\Gamma }$$: Shape function
ν: Kinematic viscosity (μ/ρ)(Pa.s)
Ø: Nanoparticle volume fraction (%)
ω: Angular velocity (rad/sec)
Ω: Dimensionless angular velocity
β: Thermal expansion coefficient (K^-1^)
ρ: Density (kg/m^3^)
σ: Electrical conductivity

## Subscripts

c: Cold
bf: Base Fluid (pure)
sp: Solid particle
na: Nanofluid
so: Conductive solid cylinder

## Abbreviations

Cond.: Conduction
Conv.: Convection
